# Diminished appetitive startle modulation following targeted inhibition of prefrontal cortex

**DOI:** 10.1038/srep08954

**Published:** 2015-03-10

**Authors:** René Hurlemann, Stephan Arndt, Thomas E. Schlaepfer, Juergen Reul, Wolfgang Maier, Dirk Scheele

**Affiliations:** 1 Department of Psychiatry, University of Bonn, 53105 Bonn, Germany; 2 Division of Medical Psychology, University of Bonn, 53105 Bonn, Germany; 3 Department of Psychiatry & Behavioral Medicine, The Johns Hopkins Hospital, Baltimore, Maryland, 21287, USA; 4 Beta Clinic, 53227 Bonn, Germany; 5 German Center for Neurodegenerative Diseases (DZNE), 53175 Bonn, Germany

## Abstract

From an evolutionary perspective the startle eye-blink response forms an integral part of the human avoidance behavioral repertoire and is typically diminished by pleasant emotional states. In major depressive disorder (MDD) appetitive motivation is impaired, evident in a reduced interference of positive emotion with the startle response. Given the pivotal role of frontostriatal neurocircuitry in orchestrating appetitive motivation, we hypothesized that inhibitory transcranial magnetic stimulation (TMS) would reduce appetitive neuromodulation in a manner similar to MDD. Based on a pre-TMS functional MRI (fMRI) experiment we selected the left dorsolateral and dorsomedial prefrontal cortices as target regions for subsequent sham-controlled inhibitory theta-burst TMS (TBS) in 40 healthy male volunteers. Consistent with our hypothesis, between-group comparisons revealed a TBS-induced inhibition of appetitive neuromodulation, manifest in a diminished startle response suppression by hedonic stimuli. Collectively, our results suggest that functional integrity of left dorsolateral and dorsomedial prefrontal cortex is critical for mediating a pleasure-induced down-regulation of avoidance responses which may protect the brain from a depressogenic preponderance of defensive stress.

Among the core defensive responses within the human behavioral repertoire is the acoustic startle reflex. The magnitude of this reflex is tightly regulated by aversive and appetitive motivational systems, with the presentation of unpleasant foreground stimuli potentiating and pleasant ones diminishing the startle magnitude, respectively^1^. Consistent with this motivational control of avoidance responses are observations of disrupted appetitive neuromodulation in patients with major depressive disorder (MDD), evident in a reduced interference of positive emotion with the startle response^2^, a noxious preponderance of defensive stress^3^,^4^,^5^, and decreased approach-related behavior^6^,^7^.

MDD is currently ranked third worldwide in disease burden and is expected to rank first in high-income countries in 2030^8^. As many as a third of MDD patients suffer from treatment-refractory depression (TRD). To help those with TRD, a variety of novel brain stimulation techniques have emerged, including transcranial magnetic stimulation (TMS) over the left dorsolateral prefrontal cortex (dlPFC)^9^. Recent meta-analyses have documented response rates of 29.3% with TMS compared to 10.4% with sham treatment in MDD^10^.

Further support for the rationale to apply TMS for modulating dlPFC function comes from studies in healthy individuals. Compelling evidence has accrued that TMS over the dlPFC influences, and interacts with various facets of emotion and motivation. For instance, TMS-induced inhibition of the left dlPFC has been shown to induce behavioral biases towards increased reward responsiveness^11^,^12^,^13^ and enhance both reactive and proactive types of aggression^14^. In contrast, TMS-induced disruption of the right dlPFC diminished subjects' willingness to build a favorable reputation^15^, provoked risk-taking behavior^16^ (but see Ref. 17), and increased the probability of utilitarian moral judgments^18^. While these studies strongly implicate the dlPFC in a top-down regulation of emotion and motivation, less is known about its modulatory impact on mood. Whereas early studies found decreased happiness ratings following excitatory stimulation of the left dlPFC^19^,^20^, more recent studies either failed to detect TMS-induced mood changes^21^,^22^ or measured them only after long-term treatment^23^. On the neural level, excitation of the right dlPFC attenuated right amygdalar responses to negative emotional stimuli in healthy females^24^, adding support to the hypothesis that TMS-induced focal changes in dlPFC activity exert distant modulatory effects within subcortical regions. Consistent with this hypothesis are intriguing findings that TMS over different prefrontal areas alters striatal dopamine (DA) release^25^,^26^,^27^, which is known to substantially contribute to appetitive motivational processes^28^.

Left dlPFC metabolic hypoactivity has been linked to anhedonia in patients with MDD^29^ and thus supports the therapeutic rationale for using excitatory TMS over this region. Less is known, however, about the mechanistic role of the left dlPFC in appetitive neuromodulation. To address this question, the present study was designed to model a deficit in pleasure-induced startle response suppression in healthy subjects by compromising left dlPFC function with inhibitory TMS. Based on the results of a pre-TMS functional MRI (fMRI) localizer task we selected the left dlPFC and an adjacent area, the dorsomedial prefrontal cortex (dmPFC), as target regions for subsequent sham-controlled inhibitory theta-burst TMS (TBS) in 40 healthy male volunteers. Our strategy was to use a prolonged intermittent TBS protocol known to produce long-lasting inhibitory after-effects^30^, before subjects were tested on the emotional startle paradigm and a complementary cognitive emotion judgment task. Given the pivotal role of frontostriatal neurocircuitry in orchestrating top-down influences on value assignment to hedonic stimuli^31^, we hypothesized that TBS-induced dlPFC dysfunction would particularly affect striatal responses. We therefore predicted that our intervention would specifically interfere with a startle reflex attenuation by positive emotion, thus modeling a lack of appetitive neuromodulation that constitutes a core characteristic of the depressive phenotype.

## Results

### Results of the fMRI localizer task

**Figure 1:**
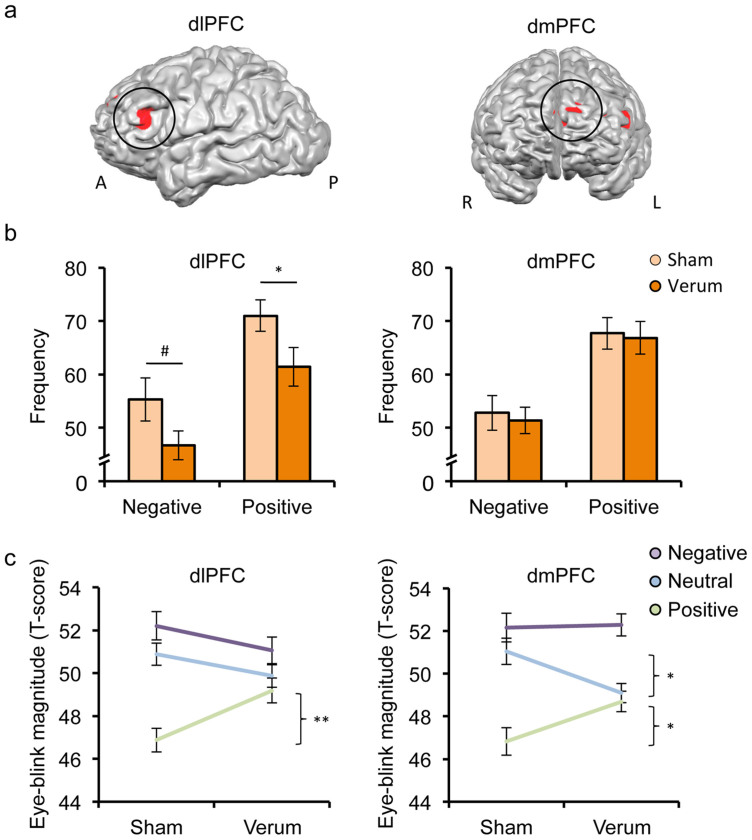
The evaluation of emotional stimuli compared to neutral ones elicited robust responses in the left dorsolateral and dorsomedial prefrontal cortex (dlPFC and dmPFC). The activations are illustrated as 4 mm spheres around the peak coordinates on the surfaces of the outer grey matter boundary (a). An inhibitory theta burst stimulation (TBS) of the left dlPFC but not of the left dmPFC reduced the frequency of emotion-startle interactions, underscoring the focality of our fMRI-guided intervention. This frequency was defined as the percentage of trials entailing a larger startle magnitude when negative stimuli were presented and a smaller magnitude when positive stimuli were shown (relative to the neutral category) (b). Disruption of the left dlPFC or dmPFC reduced the magnitude of appetitive startle modulation, while leaving a startle potentiation by negative emotion unaffected (c). Error bars indicate the standard error of the mean (SEM). Abbreviations: A, anterior; dlPFC, dorsolateral prefrontal cortex; dmPFC, dorsomedial prefrontal cortex; L, left; P, posterior; R, right; **P < 0.01; *P < 0.05; ^#^*P* < 0.10.

The arousal-based evaluation of emotional relative to neutral stimuli elicited activations in a broad neurocircuitry involving prefrontal and cingulate cortices, limbic areas as well as parietal and occipital regions (cf. Supplemental Table S2). In the present study, we focused on two dorsal prefrontal regions (dlPFC and dmPFC; cf. Fig. 1a) for two main reasons: first, the vast majority of studies examining potential antidepressant TMS effects targeted prefrontal regions^9^,^10^, and second, the limited depth effects of TMS do not allow to directly probe the functional integrity of subcortical areas.

### Results of the cognitive emotion judgment task

A repeated measures analysis of variance (ANOVA) with treatment (sham vs. verum) as between-subject factor, target region (dlPFC vs. dmPFC), category (negative, neutral, positive) and measurement (pre vs. post) as within-subject factors and the valence ratings as dependent variable revealed a main effect of category (*F*_(2,76)_ = 918.77, *P* < 0.01, η^2^ = 0.96), but no main or interaction effect of treatment (all *P*s > 0.28; cf. Tables 1 and 2). Likewise, a repeated measures ANOVA with the arousal ratings as dependent variable also yielded a main effect of category (*F*_(2,76)_ = 292.14, *P* < 0.01, η^2^ = 0.89), but no main or interaction effect of treatment (all *P* values > 0.33). Thus, our TBS protocol did not alter arousal or valence ratings obtained during cognitive emotion judgments.

### Results of the emotion-modulated startle response task

To examine the baseline startle response and its habituation, we computed a repeated measures ANOVA with the raw startle response magnitudes from the interstimulus intervals as dependent variable and six time intervals (each with three startle probes) and the target region (dlPFC vs. dmPFC) as within-subject factors. While we observed a clear decline of the startle magnitude across time (*F*_(2.69,91.44)_ = 11.83, *P* < 0.01, η^2^ = 0.26, cf. Supplemental Figure S1), no significant treatment difference or treatment x time interaction was evident (all *P*s > 0.23). In an exploratory analysis, we also *t*-standardized the baseline startle magnitudes to reduce the high inter-individual variance in the startle responses, but we still did not find significant main or interaction effects of the treatment (all *P*s > 0.46).

A main effect of category (*F*_(2,76)_ = 29.14, *P* < 0.01, η^2^ = 0.43) across target regions and treatment demonstrated the expected emotion modulation of the startle response with concomitant presentation of negative or positive stimuli potentiating or diminishing the response, respectively. However, we also found a significant interaction between treatment and category (*F*_(2,76)_ = 6.13, *P* < 0.01, η^2^ = 0.14). A comparison of the effect sizes indicated that the emotion modulation in the verum group (*F*_(2,38)_ = 8.01, *P* < 0.01, η^2^ = 0.30) was almost only half as large as in the sham group (*F*_(2,38)_ = 26.01, *P* < 0.01, η^2^ = 0.58). Notably, after dlPFC stimulation the frequency of appetitive startle modulation was reduced, evident in a lower percentage of startle responses with a smaller magnitude during the presentation of positive stimuli compared to neutral ones (*t*_(38)_ = 2.07, *P* = 0.046, Cohen's *d* = 0.67, cf. Fig. 1b). There was also a trend for a decreased impact of negative information (i.e. a reduced frequency of startle responses with a larger magnitude during the presentation of negative stimuli compared to neural ones; *t*_(38)_ = 1.75, *P* = 0.09, Cohen's *d* = 0.57). No such effects were evident after dmPFC stimulation (all *P* values > 0.73). Concerning the eye-blink magnitude (T scores), post hoc unpaired t-tests revealed that the modulation of the startle magnitude in response to positive emotion was impaired following verum stimulation of both target areas (dlPFC: *t*_(38)_ = 2.87, *P* < 0.01, Cohen's *d* = 0.93; dmPFC: *t*_(38)_ = 2.32, *P* = 0.03, Cohen's *d* = 0.75, cf. Fig. 1c). Put differently, the frequency or penetrance of appetitive as well as aversive neuromodulation was diminished after dlPFC stimulation, whereas its intensity was affected after both dlPFC and dmPFC stimulation, resulting in a selective suppression of appetitive neuromodulation. There were no other significant differences (all *P* values > 0.05), with the exception of a lower startle magnitude in the neutral category after verum stimulation of the dmPFC (*t*_(38)_ = −2.55, *P* = 0.02, Cohen's *d* = 0.83). In conclusion, inhibition of the left dlPFC (and to a lesser extent also the dmPFC) attenuated the emotional modulation of the startle response particularly for positive stimuli.

## Discussion

The rationale of the present study was to test whether fMRI-guided functional lesions of prefrontal cortex subregions would interfere with a pleasure-induced startle response suppression in healthy subjects. Our pre-TBS fMRI results revealed that the arousal assessment of stimuli subsequently used as emotional primers in the startle task evoked robust left-hemispheric responses in both dlPFC and dmPFC. Under sham stimulation, we observed the expected emotion modulation of the startle response, with negative foreground stimuli potentiating and positive ones reducing the startle magnitude. This pattern of results has been interpreted as evidence for motivational priming^1^. According to this view, aversive pictures may prime the defensive motivational system and subsequently lead to stronger protective responses including a facilitation of the startle reflex. Likewise, pleasant stimuli may engage the appetitive motivational system and therefore inhibit defensive responses. Our results show that inhibitory TBS of both target regions selectively diminished an appetitive modulation of the startle magnitude in the absence of changes in mood or cognitive emotion judgments.

Thus, to some extent, these findings mimic the aberrant emotional startle response profile exhibited by MDD patients, suggesting that inhibitory TBS over prefrontal cortex subregions can model distinct psychophysical abnormalities of the depressive phenotype in the healthy. Consistent with current perspectives that depression is characterized by an emotional context insensitivity^32^, a blunted startle response modulation has been reported for MDD patients^2^,^33^,^34^,^35^,^36^ (but see Ref. 37) as well as for patients with a current anxiety disorder and a co-morbid depressive episode^38^. This aspect is reflected in our findings of a reduced frequency of both appetitive and aversive neuromodulation following focal disruption of the left dlPFC but not of the adjacent dmPFC, underscoring a pivotal role of the former in integrating bottom-up signals of emotional arousal. This valence-independent but focally restricted effect of inhibitory TBS on the arousal-related penetrance of emotion-startle interactions contrasts sharply with the observed deficit in startle magnitude modulation by hedonic stimuli - a valence-specific effect that occurred in response to inhibition of either the left dlPFC or dmPFC and cannot be explained by a disturbed integration of ascending arousal signals. Intriguingly, however, disruption of the left dlPFC has not only been shown to produce circumscribed cortical effects by inducing local decreases in metabolic activity^39^ but it also exerts more distant subcortical effects extending to the striatal reward system^25^,^26^,^27^,^40^, which may account for our findings.

Accumulating evidence implicates the left dlPFC - alone or in concert with adjacent medial regions^41^ - in representing motivational value in top-down control processes^42^. This is consistent with studies showing that excitation of the left dlPFC facilitates memory retrieval of positively valenced information^43^. While from a mechanistic perspective a TBS-induced focal dysfunction of the left dlPFC may be entirely sufficient to reduce an appetitive startle modulation, there is, however, substantial support for the assumption that inhibition of this target region is not limited to the cortex *per se* but propagates to frontostriatal neurocircuitry, thus compromising striatal responses to hedonic stimuli. For instance, animal lesion studies have shown that functional integrity of the striatum is essential for enabling a pleasure-induced down-regulation of the startle response^44^, and multiple lines of evidence converge on suggesting that dlPFC signals control striatal activity in a top-down regulative manner, thus adjusting value assignment to hedonic states and contexts^31^. Further supportive evidence comes from pharmacological challenges showing that the dopaminergic antagonist haloperidol blocks the otherwise robust startle reflex attenuation by dark-to-light transitions in rodents^45^, whereas in humans, homozygosity for the less active catechol-O-methyltransferase (COMT) gene variant, which confers increased striatal dopamine availability, is associated with an increased startle response inhibition by hedonic stimuli^46^.

In the present study, inhibitory TBS over the left dlPFC attenuated an appetitive startle modulation as did disruption of the left dmPFC, suggesting the latter might be an additional treatment target for anti-depressant excitatory TMS (see also Ref. 47). From a clinical perspective, patients with dmPFC lesions are indeed at high risk for developing severe depression compared with other brain injury groups^48^. Consistent with this increased vulnerability, a meta-analysis of voxel-based morphometry studies has confirmed extensive dmPFC gray matter deficits in MDD patients^49^. Intriguingly, a recent clinical trial involving TRD patients revealed marked symptom improvements in a subsample of patients with preserved hedonic function after TMS of the left dmPFC^50^. Our findings not only propose the dmPFC as a promising target for clinical studies, but also strongly inform the potential of TBS as a robust treatment regimen. The much shorter duration of TBS compared to classic TMS protocols may be a particular advantage and lead to improved rates of therapeutic adherence. A recent randomized sham-controlled study demonstrated that active theta-burst stimulation is a well-tolerated form of TMS and has promising antidepressant efficacy, particularly in depressed subjects within a certain range of treatment refractoriness^51^.

The observed dissociation of modulatory effects between tasks, with inhibitory TBS interfering with the influence of emotion on the startle response but not altering the cognitive evaluation of emotion, resembles the pattern of findings often observed in studies of emotional reactivity in MDD patients using the emotion-modulated startle paradigm^2^,^33^,^34^,^35^,^36^, as well as results from experiments using pharmacological probes^52^. This obvious disparity prompted some authors to suggest that aberrant emotion-startle interactions may be a much more sensitive psychophysical index of the emotional response deficits linked to the core pathophysiology of depression than cognitive self-report ratings^36^. Our results also resonate well with studies identifying distinct neural circuitries subserving the influence of emotion on the startle response and the cognitive evaluation of emotion^53^. The observed lack of inhibitory TBS effects on cognitive emotion ratings cannot be attributed to the temporal kinetics of TBS functional after-effects since these judgments preceded the startle paradigm and the prolonged intermittent TBS protocol used in our study has been shown to reliably produce depression-like plasticity for at least 60 minutes^30^. Surprisingly, disruption of the dmPFC, but not the dlPFC, also led to a significantly reduced startle magnitude during the presentation of neutral foreground stimuli. Since inhibitory TBS had no effect on the baseline startle response *per se*, the most plausible explanation for this finding is an emotional shift induced by dmPFC inhibition.

Regarding potential limitations of our study, we note that only male volunteers were included, which may limit the generalizability of our findings to both sexes. Likewise, it has been demonstrated that the modulatory influence of emotional stimuli on the startle reflex may vary as a function of the participants' age, with older adults potentially exhibiting altered emotion-startle interactions^54^. Thus, the effects of inhibitory TBS as documented by the present study cannot necessarily be extended to older populations. In addition, experimental TMS effects are often moderated by state anxiety or mood^55^. However, we can rule out any nonspecific contributions from these parameters, since there were neither pre-treatment nor post-treatment differences in state anxiety or mood between the sham and verum treated group (cf. Supplemental Tables S3 and S4). Furthermore, consistent with other TMS studies targeting the left dlPFC^56^,^57^, we observed no discrepancies in attentional performance. Moreover, we controlled for possible placebo effects by using a placebo coil that produced a slight sensory stimulation. Related to this, we note that subjects were unaware of whether they had received verum or sham treatment, and pleasantness ratings also indicate no between-group differences (cf. Supplemental Information).

In conclusion, we here provide the first evidence that fMRI-guided disruption of the left dlPFC or dmPFC in healthy subjects can induce malfunctioning of the appetitive motivational system, evident in a diminished suppression of the startle magnitude by hedonic stimuli. Our findings thus suggest that prefrontal functional integrity is critical for mediating a down-regulation of avoidance responses by pleasure signals, thus protecting the brain from a depressogenic preponderance of defensive stress.

## Methods

### Participants

Forty-one healthy, non-smoking, heterosexual, male adults (mean age ± SD = 24.12 ± 3.66 years) participated in the present study. All subjects were recruited from the University of Bonn and gave written informed consent, which was approved by the Institutional Review Board (IRB) of the Medical Faculty of the University of Bonn. All experimental protocols and procedures were conducted in accordance with the IRB guidelines for experimental testing and were in compliance with the latest revision of the Declaration of Helsinki.

Subjects displaying fewer than 50% satisfactory blink responses in the startle paradigm (verum group, n = 1) were excluded. All remaining participants (verum group: n = 20, mean age ± SD = 23.85 ± 3.25 years; sham group: n = 20, 24.45 ± 4.16 years) showed normal cognitive performance and were free of current and past physical or psychiatric illness, as assessed by medical history and a Structured Clinical Interview for DSM-IV axis I (SCID-I) and axis II disorders (SCID-II). Notably, there were no *a priori* differences between the verum and sham treated groups regarding age, education, and pre-treatment neuropsychological parameters (all *P* values > 0.05; for details see Supplemental Table S1).

### Experimental tasks

#### fMRI localizer task

To identify the anatomical target regions for subsequent inhibitory TBS, all participants initially underwent fMRI scanning on a functional localizer task requiring them to rate the emotional intensity (arousal) of negative, neutral and positive pictures selected from one of two similar stimulus sets (A and B). Neural responses associated with this arousal-based evaluation (contrast: negative and positive > neutral) were analyzed using BrainVoyager QX^58^. Data were acquired with a 1.5 Tesla Siemens Magnetom Espree MRI system (Siemens, Erlangen, Germany). Details on the task, fMRI procedure and analyses are reported in the Supplemental Information section. After fMRI scanning, subjects were randomly assigned to either verum or sham TBS, with the first of two subsequent TBS sessions commencing three months after fMRI data acquisition.

#### Cognitive emotion judgment task

Immediately after each TBS session, subjects were exposed to a cognitive emotion judgment task. Specifically, they used a 9-point self-assessment manikin (SAM) scale^59^ to rate the arousal (1, calm; 9, excited) and valence (1, negative; 9, positive) of picture set A or B. Each picture set contained 30 negative, 30 neutral and 30 positive stimuli carefully selected from the International Affective Picture System (IAPS)^59^. The order of picture sets was balanced, i.e. if set A was shown before the TBS procedure, the post-TBS rating was completed using set B. Subsequently, all subjects were submitted to the startle response task.

#### Emotion-modulated startle paradigm

During this task, participants were exposed to acoustic startle probes presented either alone or paired with a picture. The paradigm featured 20 negative, 20 neutral and 20 positive pictures of the same picture sets (A and B) used in the fMRI task three months before. The pictures were presented for 5 s and were shown in a pseudo-randomized order. The startle stimulus consisted of a single 50-ms burst of white noise (100 dB) with nearly instantaneous rise and was delivered binaurally via headphones during 60% of the pictures (i.e. 12 from each category) at 2–4 s after picture onset. A 70-dB white noise background was present throughout the experiment. Facial electromyographic (EMG) activity was recorded from two Ag/AgCl electrodes placed over the orbicularis oculi muscle below the left eye^60^. A ground electrode was placed behind the subjects' left ear. A commercial system (Contact Precision Instruments, Cambridge, MA) was used for stimulus delivery and psychophysiological recordings. In addition, 18 of 59 interstimulus intervals were accompanied by startle probes to reduce predictability. To account for early habituation, the experiment started with the presentation of five startle probes in 2-s intervals with no picture and five startle probes during the presentation of a neutral picture. The facial EMG signal was digitized at a rate of 1000 Hz and amplified with a high-pass filter of 30 Hz and a low-pass filter of 500 Hz. EMG data were rectified and smoothed by a 4-point moving average. Startle eyeblink reflex was calculated as the difference between the maximum increase of EMG activity in a time interval between 20 and 100 ms after startle probe onset and the mean EMG of the 50-ms baseline directly preceding the onset. All EMG data were z-transformed within-subject and then converted into *T*-scores to reduce between-subjects variability and skew. The frequency of emotion modulation was calculated as the proportion of z values smaller than zero (positive category) or larger than zero (negative category) relative to the number of all valid startle trials (all startle trials minus artifacts). The EMG recordings were visually inspected, and trials with excessive noise were excluded from further analysis (overall 9% of all trials). Trials with no perceptible eye-blink reflex were assigned a magnitude of zero and included in the analysis (overall 8% of all trials). Subjects displaying fewer than 50% satisfactory blink responses in the paradigm (verum group, n = 1) were excluded.

#### Theta-burst TMS protocol

We applied a randomized, placebo-controlled, between-group (sham vs. verum TBS) design, with all participants undergoing two sessions of TBS, i.e. one with the dlPFC and another one with the dmPFC as target region, in a balanced order. Assignment of the target region was balanced with respect to treatment (sham vs. verum TMS) and picture set (A vs. B). TBS was applied using a Magstim Super Rapid 2 (The Magstim Company Ltd, Whitland, U.K.) and a figure-of-eight TMS coil (air film double 70 mm coil). For the sham treatment, a placebo coil (double 70 mm) was used that provides slight sensory stimulation and discharge noise, however, without stimulating cortical tissue. We administered a prolonged intermittent theta-burst protocol which consisted of bursts containing 3 pulses at 50 Hz repeated at 5 Hz. The protocol lasted 390 s (40 cycles with a total of 1200 pulses). This protocol has been previously shown to produce long-lasting inhibitory effects^30^,^61^. Stimulation intensity was set at 80% of the individual active motor threshold (mean active motor threshold: 37.4% of maximum stimulator output, minimum 31%, maximum 53%).

A frameless stereotactic system (BrainVoyager TMS Neuronavigator system; Brain-Innovation, Maastricht, The Netherlands) was used to ensure precise coil positioning^62^. The target sites were determined as the Talairach coordinates of those areas in the fMRI group analysis which exhibited the most robust activation for the contrast [Emotional > Neutral] (dlPFC: −40, 28, 24; dmPFC: −1, 52, 33). These normalized stereotaxic coordinates were back-transformed to the individual subject's brain coordinates in native space by reversing the native-to-Talairach transformation procedure. Then, TMS fMRI guidance was based on data in AC–PC space (rotating the cerebrum into the anterior commissure – posterior commissure plane). The coil positioning was supported by a rack and the coil was held tangentially to the skull with the coil handle oriented perpendicular to the middle (dlPFC) or superior (dmPFC) frontal gyrus. The distance between the center of the coil and target point was kept as small as possible.

## Author Contributions

R.H. and D.S. designed the experiments; S.A. and D.S. conducted the experiments; R.H. and D.S. analyzed the data; J.R. contributed new reagents/analytic tools; R.H., S.A., T.E.S., W.M. and D.S. wrote the paper. All authors read and approved the manuscript in its current form.

## Supplementary Material

Supplementary Information Supplementary Information

## Figures and Tables

**Table 1:** Valence and arousal ratings in the dlPFC session

	Verum (n = 20) Mean (SD)	Sham (n = 20) Mean (SD)	*t*	*P*
Valence				
Negative pre	2.37 (0.52)	2.36 (0.69)	−0.08	0.93
Negative post	2.40 (0.57)	2.45 (0.65)	0.24	0.81
Neutral pre	5.01 (0.78)	5.13 (0.32)	0.63	0.53
Neutral post	5.05 (0.24)	5.01 (0.42)	−0.37	0.72
Positive pre	6.95 (0.62)	7.09 (0.76)	0.60	0.56
Positive post	6.92 (0.58)	6.85 (0.75)	−0.32	0.75
Arousal				
Negative pre	6.07 (1.11)	6.22 (1.11)	0.44	0.66
Negative post	6.15 (1.36)	6.23 (1.06)	0.21	0.84
Neutral pre	2.95 (0.91)	3.03 (1.07)	0.23	0.82
Neutral post	2.95 (0.92)	3.12 (1.19)	0.52	0.60
Positive pre	5.55 (0.98)	5.60 (1.30)	0.15	0.88
Positive post	5.76 (1.02)	5.61 (1.41)	−0.40	0.70

*Notes.* Pre, before the inhibitory theta burst stimulation; post, after the inhibitory theta burst stimulation.

**Table 2:** Valence and arousal ratings in the dmPFC session

	Verum (n = 20) Mean (SD)	Sham (n = 20) Mean (SD)	*t*	*P*
Valence				
Negative pre	2.27 (0.52)	2.16 (0.59)	−0.58	0.57
Negative post	2.38 (0.51)	2.31 (0.60)	−0.35	0.73
Neutral pre	5.07 (0.32)	5.15 (0.44)	0.61	0.54
Neutral post	5.06 (0.24)	5.15 (0.46)	0.81	0.43
Positive pre	6.88 (0.57)	7.06 (0.75)	0.86	0.40
Positive post	6.84 (0.51)	7.01 (0.76)	0.85	0.40
Arousal				
Negative pre	6.23 (1.01)	6.39 (0.86)	0.53	0.60
Negative post	6.17 (1.23)	6.30 (1.11)	0.35	0.73
Neutral pre	2.86 (0.92)	3.18 (1.19)	0.97	0.34
Neutral post	2.98 (1.08)	3.35 (1.30)	0.98	0.34
Positive pre	5.61 (1.04)	5.64 (1.41)	0.09	0.93
Positive post	5.65 (1.12)	5.86 (1.50)	0.51	0.61

*Notes.* Pre, before the inhibitory theta burst stimulation; post, after the inhibitory theta burst stimulation.
