# Supplementary material for: Diminished appetitive startle modulation following targeted inhibition of prefrontal cortex
Source: Sci Rep. 2015 Mar 10;5:8954. doi: 10.1038/srep08954 (PMC4354029; doi:10.1038/srep08954)
Supplement: Supplementary Information [file srep08954-s1.doc]

*Supplemental Information*

**Diminished appetitive startle modulation following targeted**

**inhibition of prefrontal cortex**

René Hurlemann, Stephan Arndt, Thomas E. Schlaepfer,

Juergen Reul, Wolfgang Maier, and Dirk Scheele

***Subjects***

*Sample characteristics*

Subjects were recruited by local advertisement at the University Bonn and provided written informed consent before study enrollment. Forty-one healthy heterosexual male volunteers participated in the experiment. Subjects displaying fewer than 50% satisfactory blink responses in the startle paradigm (verum group, n = 1) were excluded. All participants were free of current and past physical or psychiatric illness, as assessed by medical history and a Structured Clinical Interview for DSM-IV axis I (SCID-I) and axis II disorders (SCID-II). All participants were naive to prescription-strength psychoactive medication and had not taken any over-the-counter psychoactive medication in the past 4 weeks. Participants were asked to maintain their regular bed and wake times and to abstain from caffeine and alcohol intake on the days of the experiment. Tobacco smokers were excluded from participation. Verum and sham treated subjects showed no *a priori* differences regarding age, education and pre-treatment neuropsychological performance (all *P* values > 0.05; for details see **Supplemental Table S1**). To control for potentially confounding effects of the TMS treatment on state anxiety and mood, all subjects completed the State-Trait Anxiety Inventory (STAI) ^1^ and the Positive and Negative Affective Scale (PANAS) ^2^ immediately before the TMS and after the experimental task. Furthermore, all subjects completed the d2 Test of Attention (Aufmerksamkeits- und Belastungstest d2) ^3^ after the experimental task. Three repeated-measures analyses of variance (ANOVA) with ‘measurement’ (before and after the experiment) and ‘target area’ (dlPFC vs. dmPFC) as within-subjects factors, ‘treatment’ (verum vs. sham) as between-subjects factor, and ‘state anxiety’, ‘positive affect’ or ‘negative affect’ as dependent variables revealed no significant interaction effect of the treatment (all *P* values > 0.23). We only observed a main effect of measurement for positive affect (*F*_(1, 38)_ = 35.08, *P* < 0.01, ^η2^ = 0.48) indicating that across treatments and target areas the positive affect was diminished after the experiment. There was also no significant difference in the d2 attention performance between the sham and verum stimulation of the dlPFC. After stimulation of the dmPFC, the d2 attention performance was significantly decreased in the sham group. However, if we took the baseline performance into consideration (i.e. calculated the difference between the attention performance in the screening session and after the experiment) no significant difference remained (*t*_(28.58)_ = -1.58, *P* = 0.13). Thus, verum TMS did not influence subjective anxiety, mood ratings, or attention more than sham stimulation. After completing the task in the last session, subjects were debriefed and asked to guess whether they had received verum or sham treatment. The estimation of the received treatment was comparable between the verum and sham session (dlPFC: χ^2^_(1)_ = 3.14; *P* = 0.18; dmPFC: χ^2^_(1)_ = 2.50; *P* = 0.11), showing that the subjects were unaware of whether they had received verum or sham TMS. Importantly, we asked all subjects to rate the pleasantness of the stimulation on a scale from 1 (“very unpleasant”) to 9 (“very pleasant”) after the experiment and we did not observe any differences between the verum and sham stimulation (dlPFC: verum 4.95 ± 1.79, sham 5.45 ± 1.73, *t*_(38)_ = 0.90, *P* = 0.38; dmPFC: verum 5.20 ± 1.51, sham 5.80 ± 1.91, *t*_(38)_ = 1.10, *P* = 0.28).

*Neuropsychological screening*

To control for possible pre-treatment differences in cognitive performance, all participants completed a comprehensive neuropsychological test battery. Neuropsychological testing included the German version of the RAVLT (Rey Auditory Verbal Learning Test) ^4,5^ to assess verbal learning skills, the DST (digit-span test) derived from the revised Wechsler adult intelligence scale ^6^ to assess working memory performance, the LPS 4 (‘Leistungspruefsystem Subtest 4’) ^7^ to assess nonverbal reasoning IQ, the MWT-B (‘Mehrfach-Wortschatz-Intelligenztest Teil B’) ^8^ to assess verbal IQ based on lexical decisions, and the trail-making test (TMT) part A and B ^9^ to assess visual attention and task-switching abilities.

***Procedure and design***

All subjects were screened extensively in a first session to ensure that they fulfilled all inclusion criteria for an fMRI and TMS study. The mean interval between the two TMS sessions was 3.39 days (minimum 1 day, maximum 13 days).

*Imaging*

For the imaging paradigm two picture sets (A and B) each with 30 negative, 30 neutral and 30 positive pictures were taken from the International Affective Picture System ^10^. A pilot study involving an independent sample of eight healthy male volunteers revealed that valence (“1” = very negative, “9” = very positive) and arousal (“1” = very low, “9” = very high) ratings of both picture sets were comparable (valence mean ± S.D.: negative A 2.68 ± 0.96 and B 2.69 ± 0.91; neutral A 5.43 ± 0.66 and B 5.28 ± 0.49; positive A 6.94 ± 0.67 and B 6.82 ±0.70; arousal mean ± S.D.: negative A 5.93 ± 1.43 and B 6.10 ± 1.71; neutral A 3.38 ± 0.97 and B 2.92 ± 0.83; positive A 5.26 ± 1.13 and B 5.11 ± 0.97). The semantic contents of the pictures comprised attractive women (in one picture together with a child) and erotic heterosexual couples in the pleasant condition, household (e.g. knobs, clothespins, a screwdriver) and kitchen objects (e.g. a spoon, a cup) in the neutral condition, and attacking humans (e.g. knife and gun assaults), injured humans (e.g. an accident victim, a starving child, a burning man) and mutilated bodies in the negative condition. Using an in-house programmed script the pictures were adjusted to closely resemble each other in luminance. During the imaging, the pictures of one set were presented in random order. Stimuli were presented for 3 s on-screen and they were separated from each other by a low-level baseline period (duration between 3 and 6 s, mean 5 s), where a fixation cross was depicted in the center of the screen. All subjects were instructed to rate the arousal induced by the picture as either low, medium or high by pressing one of three buttons. Furthermore, a simple finger tapping task was used to localize the hot spot in the motor cortex for the motor threshold determination. The task consisted of four 30 s blocks in which the subjects had to press a button with their thumb. Between the blocks a fixation cross was shown and the presentation duration varied between 25 and 35 s (mean 30 s) to create jitter.

*fMRI data acquisition*

FMRI employing blood oxygenation level-dependent (BOLD) contrast was carried out on a 1.5 Tesla Siemens Magnetom Espree MRI system (Siemens, Erlangen, Germany) using a T2*-weighted echo planar imaging sequence (imaging parameters: TR = 3000 ms, TE = 50 ms, matrix size: 64 x 64, pixel size: 3 x 3 x 3 mm, slice thickness = 3.0 mm, distance factor = 10%, FoV = 210, flip angle = 90°, 35 axial slices). In addition, high-resolution anatomical images were acquired on the same scanner using a T1-weighted 3D MPRAGE sequence (imaging parameters: TR = 1660 ms, TE = 3.09, matrix size 256 x 256, pixel size 1 x 1 mm, slice thickness = 1.0 mm, FoV = 256, flip angle = 15°, 160 sagital slices).

*fMRI data analysis*

FMRI data were preprocessed and analyzed using the Brain-Voyager QX 2.3 software package with default settings (Brain Innovation, Maastricht, The Netherlands). Anatomical data were corrected for intensity inhomogenities and transformed to Talairach space ^11^. Then, a reconstruction of the cortical surface was created for both hemispheres to support TMS coil positioning by improving the visualization of the anatomical gyrification. The first five volumes of each functional time series were discarded to allow for T1 equilibration. Preprocessing steps included slice scan time correction (cubic spline interpolation), a rigid-body algorithm which rotates and translates each functional volume in 3D space in order to correct for small head movements between scans, and high-pass filtering (two cycles). Then, functional data were coregistered with the anatomical data. A general linear model (GLM) was defined for each subject that examined the neural response to the arousal evaluation of the stimuli. Specifically, separate regressors for negative, neutral, and positive pictures were designed. Each regressor was convolved with a standard model of the hemodynamic response. In a second level, random-effects analysis, the following contrasts were computed [Negative > Neutral], [Positive > Neutral], [Negative > Positive], [Negative < Positive], and [Negative and Positive > Neutral]. The resulting map of the t statistic image was thresholded at *P* < 0.05 corrected for multiple comparisons based on the false discovery rate (FDR) method.

*Transcranial magnetic stimulation (TMS) protocol*

The active motor threshold was determined by employing a maximum-likelihood parameter estimation strategy without a priori information ^12,13^. The coil was positioned over the most active spot of the motor cortex identified in the finger tapping fMRI task and the motor evoked potentials (MEP) in the right abductor pollicis brevis were measured via electromyography (MEP criterion: above 200 µV in amplitude). Apart from muscle twitches, two subjects in the sham group (two after dmPFC and two after dlPFC stimulation) and one subject in the verum group (dlPFC) reported side-effects (slight headache). All side-effects were temporary and vanished after a couple of hours.

*Experimental set-up and stimuli*

Subjects were seated approximately 100 cm in front of a computer screen and instructed to view the pictures presented on-screen and to disregard noises they might hear. All subjects used the 9-point self-assessment manikin (SAM) scale to rate the arousal (1, calm; 9, excited) and valence (1, negative; 9, positive) of picture set A or B before and immediately after the TMS. All stimuli were presented for 4 s and the subjects were informed that they had to answer within this time limit. Overall, the responses of only 2.6% of all trials were given too late. Pictures were presented in two separate runs with ten pictures of each valence category (30 pictures per run).

*Statistics*

Demographical, neuropsychological, and psychophysiological data were analyzed using SPSS 20 (SPSS Inc., Chicago, IL, USA). Quantitative behavioral data were compared by repeated measures ANOVAs. Partial eta-squared and Cohen’s d were calculated as measure of effect sizes. The assumption of sphericity was assessed with Mauchly’s test, and for significant violations Greenhouse-Geisser’s correction was applied. For qualitative variables Pearson’s chi-squared tests and Fisher’s exact tests were used. All reported *P*-values are two-tailed and *P* values of *P* < 0.05 were considered significant.

***Supplemental results***

*FMRI*

A direct comparison of the negative and positive conditions revealed that only the anterior cingulate cortex exhibited significantly stronger activations during the processing of positive stimuli (Talairach x, y, z: -1, 34, 18, *t*_(40)_ = 5.56, *P*_FDR_ < 0.05).

*Latencies of the valence and arousal ratings*

We also analyzed the reaction times for the arousal and valence ratings of the stimuli used in the startle paradigm. The reaction times of all judgments were comparable between both treatment group, except for the valence ratings of neutral and positive pictures in the dlPFC session. In these categories, the verum group was significantly slower than the sham group in the pre ratings before the TMS (neutral: verum 2.44 ± 0.48 s, sham 2.15 ± 0.37 s; *t*_(38)_ = -2.10, *P* = 0.04, Cohen’s *d* = 0.68; positive: verum 2.29 ± 0.50 s, sham 1.97 ± 0.31 s; *t*_(38)_ = -2.48, *P* = 0.02, Cohen’s *d* = 0.80).

*Startle latencies*

Startle latencies were analyzed in a repeated measures ANOVA with ‘treatment‘ as between-subjects factor (verum vs. sham) and ‘category’ (negative, neutral, or positive) and ‘target area’ (dlPFC vs. dmPFC) as within-subjects factors. There was no main effect of treatment (*P* = 0.18), but we found a threefold interaction of treatment, category and target area (*F*_(2,76)_ = 5.16, *P* < 0.01, ƞ^2^ = 0.12). Post hoc unpaired t-tests showed that only in the neutral category the latency of the startle response was reduced after verum stimulation of the dlPFC (0.057 ± 0.01s) compared to sham stimulation (0.065 ± 0.01s) (*t*_(38)_ = 2.44, *P* = 0.02, Cohen’s *d* = 0.79).

*Emotion modulation of the startle*

To further investigate the specificity of our results, we used the startle magnitudes (T scores) and calculated the contrasts ‘negative minus neutral’ and ‘positive minus neutral’ as indices of emotion modulation. The inhibitory stimulation of both the dlPFC and dmPFC decreased the positive emotion modulation index (dlPFC: *t*_(38)_ = 2.67, *P* = 0.01, Cohen’s *d* = 0.87; dmPFC: *t*_(38)_ = 2.93, *P* < 0.01, Cohen’s *d* = 0.95), but had no effect on the negative index (all *P* values > 0.14).

To examine possible repetition effects, we computed a repeated measures ANOVA with session (first vs. second) and category (negative, neutral, positive) as within-subject factors, treatment (sham vs. verum) as between-subject factor and the startle magnitude as dependent variable. There was no significant main or interaction effect of session (all *P* values > 0.05) suggesting that the repetition of the emotional startle paradigm did not confound our results.

***Supplemental References***

1 Spielberger, C. D., Gorsuch, R. L. & Lushene, R. E. *Manual for the State-Trait Anxiety Inventory*. (Consulting Psychologists Press, 1970).

2 Watson, D., Clark, L. A. & Tellegen, A. Development and validation of brief measures of positive and negative affect: the PANAS scales. *J. Pers. Soc. Psychol.* **54**, 1063-1070 (1988).

3 Brickenkamp, R. & Zillmer, E. *The d2 Test of Attention*. 1st US edn, (Hogrefe & Huber Publishers, 1998).

4 Rey, L. B. L’examen psychologique dans les cas d’encephalopathie traumatique. [The psychological examination in cases of traumatic encepholopathy]. *Archives of Psychology* **28**, 215-285 (1941).

5 Helmstaedter, C., Lendt, M. & Lux, S. *VLMT - Verbaler Lern- und Merkfähigkeitstest*. [VLMT – verbal learning and memory test]. (Beltz Test GmbH, 2001).

6 Wechsler, D. *Wechsler Adult Intelligence Scale. Administration and Scoring Manual, 3 edn*. (Psychological Corporation, 1997).

7 Horn, W. *Leistungsprüfsystem L-P-S*. [performance testing system L-P-S]. (Hogrefe, 1983).

8 Lehrl, S., Triebig, G. & Fischer, B. Multiple choice vocabulary test MWT as a valid and short test to estimate premorbid intelligenc. *Acta Neurol. Scand.* **91**, 335-345 (1995).

9 Raitan, R. M. Validity of the trail making test as an indication of organic brain damage. *Percept. Mot. Skills* **8**, 271-276 (1958).

10 Lang, P. J., Bradley, M. M. & Cuthbert, B. N. *International affective picture system (IAPS): Affective ratings of pictures and instruction manual. Technical Report A-6*. (Gainesville, FL: University of Florida, 2005).

11 Talairach, J., Tournoux, P. *Co-Planar Stereotaxic Atlas of the Human Brain*. (Thieme, 1988).

12 Awiszus, F. Fast estimation of transcranial magnetic stimulation motor threshold: is it safe? *Brain Stimul.* **4**, 58-59 (2011).

13 Silbert, B. I., Patterson, H. I., Pevcic, D. D., Windnagel, K. A. & Thickbroom, G. W. A comparison of relative-frequency and threshold-hunting methods to determine stimulus intensity in transcranial magnetic stimulation. *Clin. Neurophysiol.* **124**, 708-712 (2013).

***Supplemental Tables***

**Supplemental Table S1.** Demographics and neuropsychological performance at baseline.

|  | Verum  (n = 20)  Mean (± SD) | Sham  (n = 20)  Mean (± SD) | *t* | *P* |
| --- | --- | --- | --- | --- |
| Age (years) | 23.85 (3.25) | 24.45 (4.16) | 0.51 | 0.61 |
| Education (years) | 15.80 (2.26) | 16.30 (3.21) | 0.57 | 0.57 |
| RAVLT |  |  |  |  |
| Trial 1-5 ^1^ | 64.95 (6.42) | 62.15 (6.74) | -1.35 | 0.19 |
| Trial 6 Retention ^2^ | 13.95 (1.31) | 13.25 (1.94) | -1.33 | 0.19 |
| Trial 7 Delayed Recall ^3^ | 13.95 (1.57) | 13.45 (1.93) | -0.90 | 0.38 |
| LPS-4 ^4^ | 31.85 (3.92) | 31.00 (3.10) | -0.76 | 0.45 |
| MWT-A ^5^ | 31.15 (3.33) | 29.10 (4.32) | -1.68 | 0.10 |
| d2 ^6^ | 212.05 (41.74) | 199.35 (37.56) | -1.01 | 0.32 |
| TMT-A ^7^ | 24.20 (7.31) | 26.95 (7.72) | 1.16 | 0.26 |
| TMT-B ^7^ | 54.88 (8.96) | 55.44 (14.51) | 0.14 | 0.89 |
| Digit-span, forward ^8^ | 8.75 (1.59) | 8.75 (1.83) | 0 | 1 |
| Digit-span, backwards ^8^ | 8.65 (1.59) | 9.00 (2.08) | 0.47 | 0.64 |
| BDI ^9^ | 3.85 (4.34) | 4.25 (3.13) | 0.33 | 0.74 |
| STAI-Trait ^10^ | 41.85 (3.12) | 42.75 (4.93) | 0.69 | 0.49 |
| NEO: Openness to experience^11^ | 2.68 (0.61) | 2.91 (0.59) | 1.21 | 0.24 |
| NEO: Conscientiousness ^11^ | 3.05 (0.63) | 3.19 (0.47) | 0.79 | 0.43 |
| NEO: Extraversion ^11^ | 2.98 (0.47) | 3.11 (0.39) | 0.90 | 0.38 |
| NEO: Agreeableness ^11^ | 2.41 (0.50) | 2.37 (0.44) | -0.23 | 0.82 |
| NEO: Neuroticism ^11^ | 1.69 (0.35) | 1.68 (0.48) | -0.02 | 0.99 |

*Notes.* Verbal declarative memory performance was assessed using a German adaption of the RAVLT (Rey Auditory Verbal Learning Test) and included ^1^ learning performance across five trials (maximum possible score 75), ^2^ susceptibility to interference (maximum possible score 15), and ^3^ delayed recall (maximum possible score 15). Nonverbal reasoning IQ was assessed by the ^4^ LPS (Leistungsprüfsystem) subtest 4 (maximum possible score 40). Verbal IQ based on lexical decisions was assessed by the ^5^ MWT-A (Mehrfachwahl-Wortschatz-Intelligenz-Test Teil A) (maximum possible score 37), visual attention and concentration was assesses using the ^6^ d2 (Aufmerksamkeits- und Belastungstest d2), visual attention and task-switching was assessed using the ^7^ TMT-A and TMT-B (Trail-making test A, B) (results displayed in seconds) , working memory performance was assessed using the ^8^digit-span forward and backward test (maximum possible score 14). Depressive symptoms were assessed by the self-report ^9^ BDI (Beck´s Depression Scale, Version II), and anxiety symptoms by the ^10^ STAI (State Trait Anxiety Inventory). Personality traits were measured by the ^11^ NEO Five-Factor Inventory.

**Supplemental Table S2. Activation table for the GLM analysis for the contrast [Emotional > Neutral]**

| **Region** | **Right/left** | **Cluster size (voxels)** | ***t-score*** | **Talairach coordinates** | | |
| --- | --- | --- | --- | --- | --- | --- |
|  |  |  |  | **x** | **x** | **x** |
| **Cuneus** | L/R | 38564 | 7.95 | 11 | -98 | 18 |
| **Precentral gyrus** | R | 464 | 6.07 | 29 | -11 | 66 |
| **Middle occipital gyrus** | L | 53 | 5.60 | -37 | -89 | 9 |
| **Superior frontal gyrus** | R | 46 | 5.52 | 8 | 19 | 63 |
| **Medial frontal gyrus** | L/R | 305 | 5.47 | 2 | -14 | 72 |
| **Lingual gyrus** | L | 167 | 5.44 | -7 | -59 | 3 |
| **Superior frontal gyrus** | R | 10 | 5.29 | 38 | 37 | 30 |
| **Cingulate gyrus** | L/R | 106 | 5.28 | 2 | 19 | 42 |
| **Medial frontal gyrus** | L/R | 38 | 5.27 | 2 | 37 | 36 |
| **Inferior occipital gyrus** | R | 68 | 5.17 | 38 | -89 | -6 |
| **Middle occipital gyrus** | R | 61 | 5.15 | 35 | -89 | 6 |
| **Superior parietal lobule** | L | 77 | 5.06 | -37 | -62 | 54 |
| **Superior frontal gyrus** | L/R | 230 | 5.05 | -1 | 7 | 54 |
| **Precuneus** | R | 115 | 5.01 | 20 | -74 | 51 |
| **Cuneus** | R | 152 | 5.00 | 5 | -62 | 6 |
| **Paracentral Lobule** | L/R | 27 | 4.98 | 2 | -38 | 69 |
| **Precuneus** | R | 100 | 4.90 | 20 | -77 | 42 |
| **Superior frontal gyrus** | L/R | 38 | 4.88 | -1 | 34 | 51 |
| **Precuneus** | L | 48 | 4.82 | -28 | -74 | 18 |
| **Superior temporal gyrus** | R | 63 | 4.81 | 50 | 16 | -9 |
| **Middle occipital gyrus** | L | 29 | 4.80 | -31 | -86 | 0 |
| **Inferior frontal gyrus** | L | 12 | 4.78 | -46 | 49 | 0 |
| **Parahippocampal gyrus** | R | 25 | 4.78 | 11 | -5 | -12 |
| **Superior parietal lobule** | L | 78 | 4.77 | -25 | -71 | 45 |
| **Medial frontal gyrus** | L/R | 37 | 4.73 | -1 | 13 | 45 |
| **Inferior frontal gyrus** | R | 11 | 4.70 | 53 | 19 | 6 |
| **Precentral gyrus** | R | 14 | 4.70 | 41 | -2 | 33 |
| **Superior frontal gyrus** | L | 239 | 4.68 | -4 | 4 | 69 |
| **Middle occipital gyrus** | R | 206 | 4.63 | 26 | -83 | 12 |
| **Medial frontal gyrus** | R | 13 | 4.53 | 5 | 13 | 45 |
| **Lingual gyrus** | R | 21 | 4.42 | -13 | -53 | 0 |
| **Superior temporal gyrus** | R | 23 | 4.42 | -28 | 22 | -27 |
| **Middle temporal grus** | L | 10 | 4.39 | 59 | -2 | -12 |
| **Superior parietal lobule** | L | 25 | 4.38 | 14 | -59 | 66 |
| **Fusiform gyrus** | L | 18 | 4.36 | -40 | -44 | -15 |
| **Superior frontal gyrus** | R | 63 | 4.36 | 8 | 4 | 69 |
| **Middle frontal gyrus** | R | 19 | 4.33 | 38 | 1 | 42 |
| **Parahippocampal gyrus** | L | 41 | 4.33 | -10 | -35 | 0 |
| **Postcentral gyrus** | R | 13 | 4.31 | 38 | -32 | 66 |
| **Insula** | L | 19 | 4.27 | -25 | 16 | -9 |
| **Cuneus** | L | 18 | 4.26 | 29 | -86 | 21 |
| **Lingual gyrus** | L | 25 | 4.26 | -10 | -68 | 3 |
| **Superior frontal gyrus** | L/R | 21 | 4.25 | 2 | 22 | 57 |
| **Lingual gyrus** | L | 31 | 4.24 | -13 | -77 | 3 |
| **Middle occipital gyrus** | R | 17 | 4.23 | 26 | -95 | 9 |
| **Precuneus** | L | 66 | 4.18 | -4 | -74 | 51 |
| **Superior parietal lobule** | L | 69 | 4.17 | 29 | -59 | 48 |
| **Lingual gyrus** | R | 43 | 4.17 | -13 | -98 | -15 |
| **Inferior frontal gyrus** | L | 66 | 4.16 | -49 | 16 | 0 |
| **Middle frontal gyrus** | L | 11 | 4.14 | -40 | 28 | 24 |
| **Superior frontal gyrus** | R | 29 | 4.12 | 5 | 7 | 66 |
| **Superior frontal gyrus** | L/R | 15 | 4.10 | 2 | 7 | 48 |
| **Middle temporal gyrus** | R | 25 | 4.08 | 47 | -74 | 12 |
| **Precuneus** | R | 26 | 4.04 | 5 | -68 | 54 |
| **Superior frontal gyrus** | R | 10 | 4.03 | 11 | 7 | 69 |
| **Fusiform gyrus** | R | 17 | 4.00 | 41 | -47 | -9 |
| **Inferior parietal lobule** | L | 50 | 3.97 | -43 | -47 | 54 |
| **Precentral gyrus** | L | 30 | 3.95 | -31 | -11 | 66 |
| **Superior frontal gyrus** | L/R | 12 | 3.94 | -1 | 52 | 33 |
| **Lingual gyrus** | L/R | 11 | 3.85 | -1 | -92 | -18 |
| **Middle frontal gyrus** | R | 11 | 3.83 | 35 | 1 | 60 |
| **Medial frontal gyrus** | L/R | 13 | 3.82 | -1 | -2 | 63 |
| **Postcentral gyrus** | R | 10 | 3.66 | 50 | -20 | 57 |
| **Medial frontal gyrus** | L/R | 12 | 3.66 | 2 | 1 | 60 |

*Notes*. L, left; R, right.

**Supplemental Table S3. State measurement of anxiety, mood and attention in the dlPFC session**

|  | Verum  (n = 20)  Mean (SD) | Sham  (n = 20)  Mean (SD) | *t* | *P* |
| --- | --- | --- | --- | --- |
| STAI – pre ^1^ | 30.75 (4.71) | 32.65 (5.37) | 1.19 | 0.24 |
| STAI – post ^1^ | 32.35 (4.76) | 34.50 (5.10) | 1.38 | 0.18 |
| PANAS – positive – pre ^2^ positive | 31.10 (5.66) | 28.50 (5.60) | -1.46 | 0.15 |
| PANAS – positive – post ^2^ | 27.20 (6.75) | 26.05(7.65) | -0.50 | 0.62 |
| PANAS – negative – pre ^2^ | 10.80 (1.28) | 11.05 (1.10) | 0.66 | 0.51 |
| PANAS – negative – post ^2^ | 10.85 (1.63) | 11.00 (1.72) | 0.28 | 0.78 |
| D2 ^3^ | 229.95 (40.80) | 224.40 (33.58) | -0.47 | 0.64 |

*Notes.* State anxiety before and after the experiment was assessed using the ^1^ STAI = State Trait Anxiety Inventory. Mood before and after the experiment was assessed using the ^2^ PANAS = Positive and Negative Affect Schedule. Attention performance after the experiment was assessed using the ^3^ D2 = Aufmerksamkeits- und Belastungstest. Abbreviations: dlPFC, dorsolateral prefrontal cortex.

**Supplemental Table S4. State measurement of anxiety, mood and attention in the dm**PFC session

|  | Verum  (n = 20)  Mean (± SD) | Sham  (n = 20)  Mean (± SD) | *t* | *P* |
| --- | --- | --- | --- | --- |
| STAI – pre ^1^ | 31.90 (6.32) | 32.60 (4.36) | 0.41 | 0.69 |
| STAI – post ^1^ | 31.50 (5.09) | 33.75 (4.41) | 1.49 | 0.14 |
| PANAS – positive – pre ^2^ positive | 32.00 (6.53) | 28.30 (5.86) | -1.89 | 0.07 |
| PANAS – positive – post ^2^ | 28.10 (6.71) | 25.00 (7.89) | -1.34 | 0.19 |
| PANAS – negative – pre ^2^ | 10.95 (1.47) | 10.80 (1.01) | -0.38 | 0.71 |
| PANAS – negative – post ^2^ | 10.70 (1.49) | 10.95 (1.47) | 0.53 | 0.60 |
| D2 ^3^ | 244.15 (38.32) | 218.25 (34.95) | -2.23 | 0.03 |

*Notes.* State anxiety before and after the experiment was assessed using the ^1^ STAI = State Trait Anxiety Inventory. Mood before and after the experiment was assessed using the ^2^ PANAS = Positive and Negative Affect Schedule. Attention performance after the experiment was assessed using the ^3^ D2 = Aufmerksamkeits- und Belastungstest. Abbreviations: dmPFC, dorsomedial prefrontal cortex.

***Supplemental Figures***

**Supplemental Figure S1**


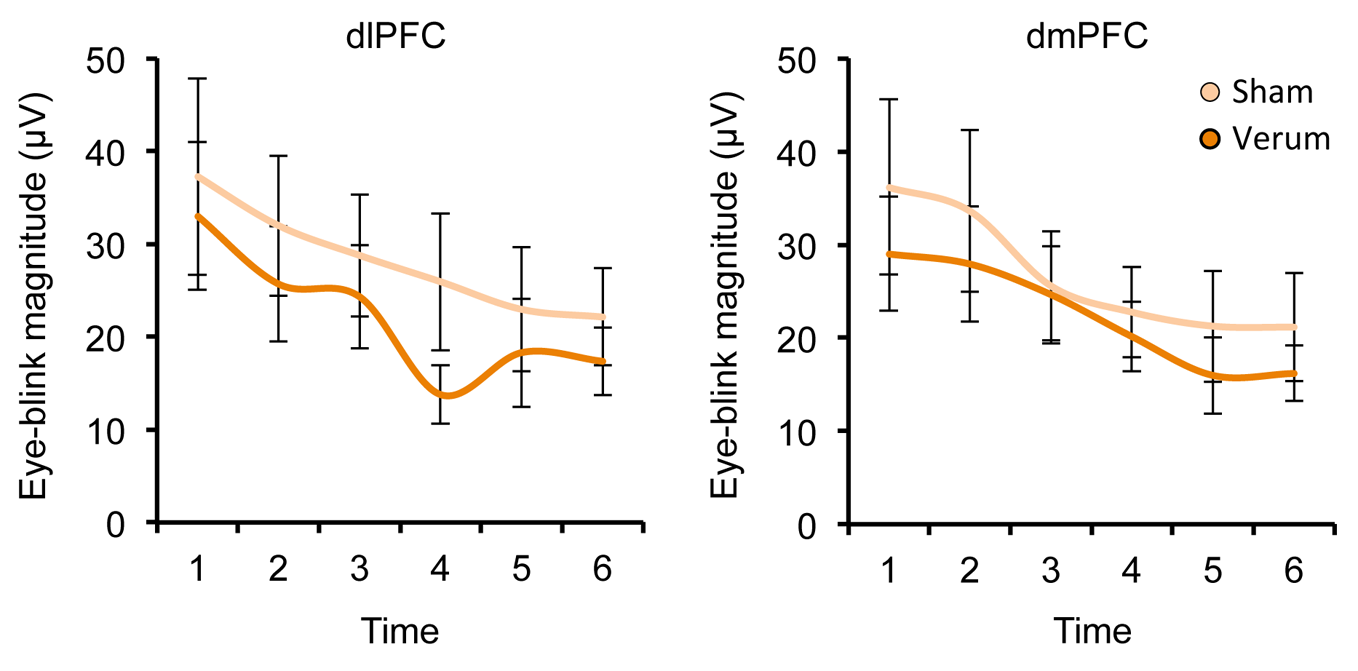


Supplemental Figure S1 illustrates the habituation of the startle response across time (three startle probes per block) in the dlPFC and dmPFC sessions. Neither the raw startle magnitude nor the decline of the magnitude over time was significantly affected by the TMS treatment. Abbreviations: dlPFC, dorsolateral prefrontal cortex; dmPFC, dorsomedial prefrontal cortex.
